# Nose Drenching

**Published:** 1871-02

**Authors:** 


### NOSE DRENCHING.

Some individuals are in the habit of drawing water through the nose into the mouth with an indefinite idea about its being advangeous in reference to catarrhal affections. The more this is done the greater craving there will be for its repetition, because cold water of fifty or sixty degrees applied to a surface whose natural temperature is near one hundred inevitably chills it; the reaction of that chill is fever and inflammation, thus drying up a surface which Nature intended should be always moist.

If warm water is used the results are not so harmful, but warm water is harsh and hard in comparison with Nature's lubricant, which is the mildest and softest possible. The internal passages and surfaces were intended to be always in a state of lubrication; if water is habitually used, it being harsh in comparison, the system learns to rely upon these artificial moistenings, and the surfaces must be dry for a greater part of the time, as it is impossible to be sniffling water up the nose every half-hour in the twenty-four, the result being a feeling of want of something whenever the parts are not sufficiently moist. All know what clots of hard substances are blown from the nose at times; these are loosened by the natural lubricants poured out under them, and pushing them off from the surface as fast as is needed, keeping the nasal passages clean and clear of obstructions and admitting a full supply of air at each breath: certainly these are important considerations; every one has felt what a delightful sense of relief there is the moment after a large clot has been discharged from the nose.

If these considerations are not sufficient to induce the abandonment of the unnatural and hurtful habit, let it be remembered that the water sniffled through the nose into the mouth carries with it whatever of the natural secretion of the nose happens to be there. Our whole nature revolts at the idea of taking into the mouth what has been discharged from the nose, and yet the snifflers do this in effect.

Very many persons are in the habit of closing the mouth and making a forcible suction of air through the nose into the mouth, the effect of which is to carry the contents of the nose into the mouth, and then spitting it out or swallowing it, instead of discharging it through the nose into the pocket handkerchief. The habit is so common, and so utterly disgusting, that one would think that it was only necessary to draw attention to it to secure its universal abandonment.

---

BUCKWHEAT CAKES AND MOLASSES for breakfast and the twenty different preparations of Rand's Sea Moss Farina as dessert for dinner, are peculiarly appropriate for cold weather, as they contain more of the warming elements than even roast beef.

FARM HOUSE MILK is in great demand in wintry times for a variety of table uses; it is furnished in all its freshness and purity by the energy and watchfulness of S. W. Canfield, President of the New Jersey and Rockland Co. Milk Association, in Fourth Avenue, opposite Stewart's, and at several other places in New York.

THE LONDON LANCET, $5 a year, reprinted by W. C. Herald, Esq., 52 John St., merits the patronage of educated physicians everywhere.

The REV. DR. JOHN LORD is delivering a course of morning lectures in Association Hall, twenty in number, historical and biographical, which, for instructiveness and absorbing interest, are not surpassed. Dr. Lord is among the very foremost of living lecturers, wide-awake in voice, gesture, enunciation, and the spirit of his subject.

N. B. — Address all business letters to Hurd & Houghton, 13 Astor Place, New York; professional to Dr. W. W. Hall, 176 Broadway, Room 8, from 10 to 2, and at No. 2 West 43d St., corner of 5th Avenue, from 3 to 4 daily.

